# Marca-Passo Cardíaco Artificial Permanente no Tratamento das Bradiarritmias Causadas pela Doença de Chagas

**DOI:** 10.36660/abc.20260180

**Published:** 2026-05-05

**Authors:** Roberto Costa

**Affiliations:** 1 Faculdade de Medicina da Universidade de São Paulo São Paulo SP Brasil Faculdade de Medicina da Universidade de São Paulo (FMUSP) São Paulo, SP - Brasil

**Keywords:** Doença de Chagas, Marca-Passo Artificial, Prevalência, Mortalidade, Fatores de Risco

Os programas governamentais, que visaram à eliminação da transmissão vetorial da Doença de Chagas, tiveram forte impacto na prevalência das bradiarritmias cardíacas secundárias a esta etiologia e, consequentemente, no número de implantes de marca-passos realizados no Brasil.^1-3^ Dados do Registro Brasileiro de Marca-passos mostram que, em 1995, dos 6.542 implantes iniciais de marca-passos informados, 30,2% se referiam a pacientes com Doença de Chagas.^4^ A proporção de pacientes com Doença de Chagas, entretanto, diminuiu com o passar do tempo e, sete anos depois, quando os dados de 2002 foram apresentados, dos 11.347 implantes iniciais reportados, a Doença de Chagas foi informada como etiologia em 25,0% dos casos.^5^ A totalização dos números dos 10 primeiros anos do presente século mostrou que a Doença de Chagas correspondeu a 17,8% dos implantes iniciais de marca-passo, caindo para 11,7% na última publicação dos dados do Registro Brasileiro de Marca-passos, referentes ao período de 2009 a 2014.^6^ Dentre os implantes iniciais de marca-passos convencionais realizados no Instituto do Coração-HC-FMUSP nos seis últimos anos (01/01/2020 a 31/12/2025), dos 2689 pacientes operados, apenas 236 (8,8%) tinham como etiologia de sua bradiarritmia a Doença de Chagas, conforme dados clínicos e demográficos de pacientes consecutivos submetidos a procedimentos para implantes de dispositivos cardíacos eletrônicos de 01/01/2020 a 31/12/2025 no Incor-HC-FMUSP.

**Figura 1:**
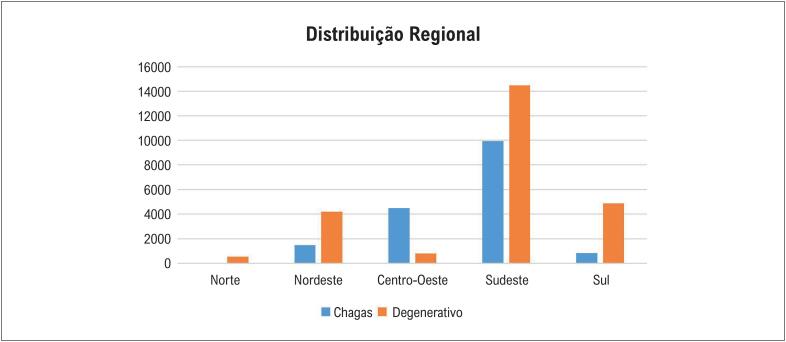
Números absolutos de implantes iniciais de marca-passo nas cinco regiões brasileiras nos anos 1995 a 2003 segundo dados do Registro Brasileiro de Marca-passos.

A análise comparativa dos dados demográficos e clínicos de 25.648 com Doença de Chagas e de 31.984 com doença degenerativa do sistema excito-condutor do coração, submetidos a implante de marca-passo convencional no período de 1995 a 2003, mostrou semelhança entre esses dois grupos de indivíduos quanto à distribuição nos sexos, com 47,8% de mulheres dentre os com Doença de Chagas e 48,5%, na doença degenerativa (p=0,186). Por outro lado, se observou significativa diferença quando se avaliou a idade média dessas duas populações, com 58,6 ±15,3 anos de idade para a Doença de Chagas e 73,5 ± 12,6, para a doença degenerativa (p=0,001). Foi observada grande diferença na prevalência dos implantes iniciais de marca-passo nas 5 regiões brasileiras, com amplo predomínio da etiologia da Doença de Chagas no Centro-Oeste, não obstante o grande número de implantes realizados no Sudeste do país (Figura 1). Embora houvesse semelhanças entre os achados eletrocardiográficos que justificaram o implante de marca-passo nessas duas populações, notou-se um maior percentual de pacientes com BAV avançado nos indivíduos com doença degenerativa; e de disfunção sinusal e de bloqueios fasciculares nos casos com Doença de Chagas.^7^

A evolução clínica dos indivíduos com Doença de Chagas submetidos a implante de marca-passo cardíaco artificial convencional, é, geralmente, muito satisfatória, sendo que a maioria dos pacientes sobrevive por muitos anos com boa qualidade de vida.^8,9^ Uma parcela considerável dos pacientes, entretanto, evolui com disfunção ventricular esquerda progressiva, insuficiência cardíaca grave, taquiarritmias, ou apresentam morte súbita. Vários estudos clínicos tentaram detectar fatores relacionados à má evolução, em particular, o modo de estimulação empregado. Pacientes com função ventricular esquerda normal ou pouco alterada no momento do implante, apresentam uma baixa probabilidade de evoluir com disfunção ventricular ou com insuficiência cardíaca, independentemente de terem sido submetidos ao implante de marca-passo ventricular ou atrioventricular.^8,9^ Por outro lado, pacientes que já apresentam disfunção ventricular esquerda, moderada ou grave, podem se beneficiar da terapia de ressincronização cardíaca (TRC), como tratamento inicial ou por mudança de modo de estimulação.^10,11^ No acompanhamento de longo prazo de pacientes com Doença de Chagas submetidos à TRC, entretanto, verificou-se menor expectativa de sobrevivência do que a de pacientes com miocardiopatia dilatada isquêmica ou não isquêmica.^11^

O seguimento de número substancial de pacientes com Doença de Chagas submetidos à estimulação cardíaca artificial permanente permitiu a detecção de preditores de morte, assim como a criação e validação um escore de risco para mortalidade baseado nos seguintes achados: disfunção ventricular direita, insuficiência cardíaca em classes funcionais III ou IV, doença renal, diâmetro atrial esquerdo > 44mm, fibrilação atrial e cardiomegalia ao Rx de tórax.^12,13^

O estudo feito por Leite et al.,^14^ teve como objetivos avaliar fatores contextuais que potencialmente influenciariam a indicação e o acesso ao implante de marca-passo convencional, assim como avaliar a mortalidade de pacientes com Doença de Chagas após um seguimento de quatro anos. Infelizmente, o pequeno número amostral do estudo dificultou a explicação de seus achados, que sugeriram que variáveis incongruentes como residir em um município com maior população ou com maior cobertura da Estratégia de Saúde da Família, justificassem o maior acesso ao implante de marca-passo em contraste com a menor disponibilidade de eletrocardiógrafos, que também esteve associada ao maior acesso a esse tipo de tratamento. Outro grave problema da amostra estudada foi a desproporção entre as perdas de seguimento no grupo de indivíduos com marca-passo e as do grupo controle, que criou um viés importante para a análise da mortalidade.
