# Supplementary material for: Marca-Passo Cardíaco Artificial Permanente no Tratamento das Bradiarritmias Causadas pela Doença de Chagas
Source: Arq Bras Cardiol. 2026 May 5;123(2):e20260180. [Article in Portuguese] doi: 10.36660/abc.20260180 (PMC13155781; doi:10.36660/abc.20260180)
Supplement: Translation

# Translation: Permanent Artificial Cardiac Pacemaker in the Treatment of Bradyarrhythmias Caused by Chagas Disease

**DOI:** 10.36660/abc.20260180i

**Authors:** Roberto Costa

**Affiliations:** 1 São Paulo SP Brazil Faculdade de Medicina da Universidade de São Paulo (FMUSP), São Paulo, SP – Brazil

**Keywords:** Chagas Disease, Artificial Pacemaker, Prevalence, Mortality, Risk Factors

Government programs aimed at eliminating vector-borne transmission of Chagas disease have had a strong impact on the prevalence of cardiac bradyarrhythmias secondary to this etiology and, consequently, on the number of pacemaker implants performed in Brazil.^1-3^ Data from the Brazilian Pacemaker Registry show that in 1995, of the 6,542 initial pacemaker implants reported, 30.2% were related to patients with Chagas disease.^4^ The proportion of patients with Chagas disease, however, decreased over time, and seven years later, when the 2002 data were presented, of the 11,347 initial implants reported, Chagas disease was reported as the etiology in 25.0% of cases.^5^ The total figures for the first 10 years of this century showed that Chagas disease accounted for 17.8% of initial pacemaker implants, falling to 11.7% in the latest publication of data from the Brazilian Pacemaker Registry, referring to the period from 2009 to 2014.^6^ Among the initial implants of conventional pacemakers performed at the Heart Institute-HC-FMUSP in the last six years (01/01/2020 to 31/12/2025), of the 2689 patients operated on, only 236 (8.8%) had Chagas disease as the etiology of their bradyarrhythmia, according to clinical and demographic data of consecutive patients undergoing procedures for implantation of electronic cardiac devices from 01/01/2020 to 31/12/2025 at Incor-HC-FMUSP.

A comparative analysis of the demographic and clinical data of 25,648 individuals with Chagas disease and 31,984 with degenerative heart disease who underwent conventional pacemaker implantation between 1995 and 2003 showed similarities between these two groups of individuals regarding sex distribution, with 47.8% women among those with Chagas disease and 48.5% in the degenerative disease group (p=0.186). On the other hand, a significant difference was observed when evaluating the average age of these two populations, with 58.6 ± 15.3 years for Chagas disease and 73.5 ± 12.6 for the degenerative disease group (p=0.001).

A significant difference was observed in the prevalence of initial pacemaker implants across the five Brazilian regions, with a strong predominance of Chagas disease etiology in the Central-West region, despite the large number of implants performed in the Southeast of the country (Figure 1). Although there were similarities between the electrocardiographic findings that justified pacemaker implantation in these two populations, a higher percentage of patients with advanced AV block was noted in individuals with degenerative disease, and of sinus dysfunction and fascicular blocks in cases with Chagas disease.^7^

**Figure 1:** Absolute numbers of initial pacemaker implants in the five Brazilian regions from 1995 to 2003, according to data from the Brazilian Pacemaker Registry.

The clinical evolution of individuals with Chagas disease undergoing conventional artificial cardiac pacemaker implantation is generally very satisfactory, with most patients surviving for many years with a good quality of life.^8,9^ However, a considerable proportion of patients develop progressive left ventricular dysfunction, severe heart failure, tachyarrhythmias, or experience sudden death. Several clinical studies have attempted to identify factors related to poor outcomes, in particular, the pacing mode employed. Patients with normal or mildly altered left ventricular function at the time of implantation have a low probability of developing ventricular dysfunction or heart failure, regardless of whether they underwent ventricular or atrioventricular pacemaker implantation.^8,9^ On the other hand, patients who already have moderate or severe left ventricular dysfunction may benefit from cardiac resynchronization therapy (CRT) as initial treatment or by changing the pacing mode.^10,11^ However, in the long-term follow-up of patients with Chagas disease undergoing CRT, a lower survival expectation was observed than that of patients with ischemic or non-ischemic dilated cardiomyopathy.^11^

The follow-up of a substantial number of patients with Chagas disease undergoing permanent artificial cardiac stimulation allowed the detection of predictors of death, as well as the creation and validation of a risk score for mortality based on the following findings: right ventricular dysfunction, heart failure in functional classes III or IV, renal disease, left atrial diameter > 44mm, atrial fibrillation and cardiomegaly on chest X-ray.^12,13^

The study by Leite et al.^14^ aimed to evaluate contextual factors that could potentially influence the indication for and access to conventional pacemaker implantation, as well as to assess the mortality of patients with Chagas disease after a four-year follow-up. Unfortunately, the small sample size of the study made it difficult to explain its findings, which suggested that incongruent variables, such as residing in a municipality with a larger population or greater coverage of the Family Health Strategy, justified greater access to pacemaker implantation in contrast to the lower availability of electrocardiographs, which was also associated with greater access to this type of treatment. Another serious problem with the studied sample was the disproportion between losses to follow-up in the group of individuals with pacemakers and those in the control group, which created a significant bias in the mortality analysis.
